# Single base substitution signatures 17a, 17b, and 40 are induced by γ-ray irradiation in association with increased reactive oxidative species

**DOI:** 10.1016/j.heliyon.2024.e28044

**Published:** 2024-03-15

**Authors:** Yuya Manaka, Rika Kusumoto-Matsuo, Yusuke Matsuno, Haruka Asai, Ken-ichi Yoshioka

**Affiliations:** a Laboratory of Genome Stability Maintenance, National Cancer Center Research Institute, Tsukiji, Chuo-ku, Tokyo, Japan; b Department of NCC Cancer Science, Graduate School of Medical and Dental Sciences, Tokyo Medical and Dental University, Yushima, Bunkyou-ku, Tokyo, Japan

## Abstract

γ-Ray irradiation induces DNA double strand breaks (DSBs) and increases the risk of cancerization. Irradiated cells usually repair DSBs directly, but accumulate replication stress-associated DSBs, increasing the risk of structural variants (SVs). Although single nucleotide variants (SNVs) are also induced, it is still unclear which SNVs are induced by γ-ray irradiation. Here, we show that single base substitution (SBS) 17a, 17b, and 40 signatures were induced by γ-ray irradiation, which is mainly SNV induction in A-T bps. While SNVs induced by genomic instability were usually associated with SVs, SNVs induced by γ-ray irradiation and the associated signatures were not. As reactive oxygen species (ROS) are a possible cause of SBS17a and 17b, ROS were induced upon γ-ray irradiation (1–8 Gy), indicating the association of ROS for the SNV induction. Thus, our results reveal that ROS-associated SNVs are increased by irradiation, and that ROS-associated SNVs are induced independently of SVs.

## 1 Introduction

Exposure to γ-ray irradiation is associated with cancerization [[1], [2], [3]]. It is generally believed that this is due to the DNA damage induced by irradiation. However, γ-ray irradiation induces many different types of DNA damage, such as DSBs, single strand breaks, and oxidative nucleotide adducts [[4], [5], [6]]. Presently, it is still unclear which type of damage is associated with cancerization and how this damage leads to mutations in cancer-driver genes. A recent study revealed that mouse embryonic fibroblast cells (MEFs) repair DSBs immediately after irradiation but subsequently accumulate replication stress-associated DSBs, thereby increasing the risk of genomic instability [7,8]. The resulting genomic instability can further increase the clonal evolution of cells mutated in the ARF/p53 pathway. These results suggest that accumulation of replication stress-associated DSBs is a primary risk factor for radiation-induced mutations.

SVs and SNVs are widely induced in association with genomic instability. SVs including chromosomal translocations are induced by erroneous DSB repair between unpaired chromosomal ends, whereas SNVs are induced by DNA polymerase errors during DNA replication [9]. Although SVs and SNVs are induced by distinct pathways, their inductions are tightly associated with genomic instability, which can lead to clonal evolution of aberrant clones, as shown in the MEF model [[10], [11], [12]]. Even in the human genome, while SNVs in normal organs generally increase with age [[13], [14], [15]] as a consequence of DNA replication errors [16,17], those in the cancer genome are more tightly associated with SVs [10], implying that SNVs in cancer cells are often induced in association with genomic instability. Supporting this argument, the SBS signatures associated with genomic instability, such as homologous recombination (HR) deficiency-associated SBS3 and DNA damage-associated SBS18, accumulate to high levels, especially around SV sites (<1 M base) [10]. SBS signature types could be altered by the environmental background, e.g., inflammation, which leads to deamination-associated signatures SBS2 and SBS13.

In the present study, we studied the SNVs induced in immortalized MEFs after γ-ray irradiation, because MEFs are a useful model to study mutational patterns [18]. Our results revealed that γ-ray irradiation is associated with the induction of SBS17a, 17b, and 40 signatures. These signatures were induced regardless of the presence of SV sites, although the presence of minor SVs cannot be excluded. As ROS is known to be induced by γ-ray irradiation, ROS were the probable cause of the induction of SBS17a, 17b, and 40 signatures.

## 2 Materials and Methods

### 2.1 Analyses of SVs and SNVs

Whole-genome sequence data were obtained from previously reported irradiated tumors and controls [19,20]. Sequencing data from the European Genome-Phenome Archive (EGA, http://www.ebi.ac.uk/ega/) were used to find SBS signatures, SVs, and SNVs. Validated SV and SNV data from MEFs that had undergone clonal evolution were obtained from a previous study [8].

The association of SVs with mutational signatures was analyzed using the SBS signatures previously identified in each type of cancer. Analyses were done when the SNV number was more than 10. Mutational signatures were evaluated with SigProfilerExtractor (https://github.com/AlexandrovLab/SigProfilerExtractor, version 1.1.4) for SNVs within 1 Mb of SV sites and those in the whole genome using default settings. The numbers of SVs and SNVs were counted in every 15, 30, and 60 Mb window. Circular representations of SVs were visualized using BioCircos.

### 2.2 Estimations of SNV numbers around SV sites under random induction

To assess biased SNV generation around SV sites, SNV distribution around these sites was compared with their expected random distribution in the whole genome. The former was estimated based on the calculation (i) below. The estimated distribution may include genomic regions in which SVs and SNVs are not detectable by whole-genome sequencing (WGS) analyses, such as centromeres.

$$\text{Expected}\;\text{value}=\frac{\text{SNV}\;\text{number}}{\text{Genome}\;\text{length}}\times \text{SV}\;\text{number}\times 2\times \text{Range}\left(1\;k-10\;M\right)–\;\left(i\right)$$

### 2.3 Assumptions of SV-dependent and -independent SNV numbers

To estimate the numbers of SV-dependent and -independent SNVs, the numbers of SVs and SNVs were counted in windows of every 15, 30, and 60 Mb. Correlations between SVs and SNVs were then determined using linear regression, in which SV-dependent and -independent SNVs were assessed from the slopes and intercepts of the regression line. SV-dependent SNV numbers per unit genome size (M base) are estimated from the slope of the regression line of SNV number versus SV number. SV-independent SNV numbers per unit genome size (M base) are estimated from the intercept of the regression line of SNVs versus SVs.

### 2.4 Cell culture and assays

Primary MEFs used in this experiment were prepared from mouse embryos in a specific pathogen-free environment at the National Cancer Center Research Institute (Tokyo, Japan) animal facility according to the institutional guidelines, and with the approval of the Japan National Cancer Center Animal Ethics Committee (B104M2-18, A364M2-18). Cervical dislocation was used for euthanasia. Cells were cultivated in Dulbecco's Modified Eagle's Medium (Nacalai Tesque) supplemented with 10% (v/v) fetal calf serum (Gibco).

DNA damage was induced by treating cells with ^137^Cs irradiation in a Gammacell 40 Exactor (Best Theratronics). A549 cells irradiated with γ-ray were used for the analyses of ROS levels. ROS levels were determined using the ROS Assay Kit-Photo-oxidation Resistant DCFH-DA (Dojindo). Immunofluorescence was observed using a confocal laser microscope (Olympus FV3000).

## 3 Results

### 3.1 Generation of SBS17a, 17b, and 40 signatures by γ-ray irradiation

**Fig. 1:**
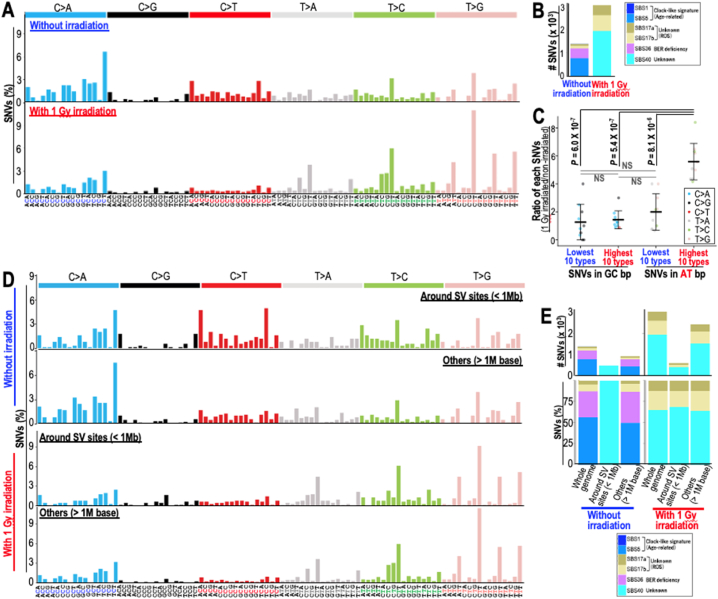
**SBS40 and 17a/b induction by radiation exposure. (A)** The spectra of mutations in immortalized MEFs with and without γ-ray irradiation were analyzed in the context of the flanking 1 bp sequence on each side and compared. **(B)** Number of SNVs associated with each SBS signature were analyzed in immortalized MEFs with and without γ-ray irradiation. **(C)** Biased SNV generation after γ-ray irradiation was analyzed by comparing the SNV signatures generated in MEFs with and without γ-ray irradiation, in which the lowest 10 and the highest 10 SNV types induced in A-T bps (T > A, T > C, and T > G) were analyzed and compared with those induced in G-C bps (C > A, C > G, and C > T). Two-tailed Welch's *t*-test was used for statistical analysis. **(D)** The spectra of mutations in immortalized MEFs occurring within 1 Mb of SV sites were compared with those occurring in other genomic regions. **(E)** Number of SNVs associated with each SBS signature were analyzed in immortalized MEFs with and without γ-ray irradiation. Those located within 1 Mb of SV sites and others were separately analyzed.

Radiation exposure is associated with increased genomic instability, which results in both SVs and SNVs [6,8,21]. Since a previous study revealed that radiation exposure is associated with increased generation of SNVs in A-T base pairs (bp) [8], we further analyzed SBS signatures after irradiation. To this end, we compared the SNVs induced in unirradiated and γ-ray irradiated MEFs that had undergone clonal evolution. As expected, we observed changes in SBS signatures after irradiation. While the age-related clock-like signature SBS5 and base excision repair (BER) deficiency-associated SBS36 signature were the major signatures in unirradiated MEFs, SBS40, 17a and 17b were the major signatures in irradiated MEFs (Fig. 1A and B). In addition, SNVs in A-T bps increased after γ-ray irradiation, i.e., especially the middle T in the XTT sequence context. In fact, while the highest 10 SNV types in A-T bps were significantly increased in irradiated MEFs (Fig. 1A), the lowest 10 SNV types in A-T bps were not, which was similar to the SNVs induced in G-C bps (Fig. 1C). These results indicate that radiation exposure is associated with SNV induction in A-T bps, which is associated with SBS40, 17a, and 17b signatures.

To address the effect of SVs on SBS signatures, we analyzed SNVs around SV sites (<1 M base) and compared them with those in other genomic regions. In irradiated MEFs, SBS signatures around SV sites were different from those in other genomic regions. While the SBS40 signature was dominant around SV sites (<1 M base), clock-like signatures SBS5 and BER deficiency-associated SBS36 were the major signatures in other genomic regions (Fig. 1D and E), which is similar to those observed in the whole genome (Fig. 1B). By contrast, SBS signatures induced by irradiation were SBS40, 17a, and 17b, which were shown regardless of their proximity to SV sites (Fig. 1E). These results indicate that radiation exposure is associated with the induction of particular types of SNVs, which are basically independent of SV induction. Given that ROS is a possible cause of SBS17a and 17b signatures, ROS might be associated with such SNV induction. Although the cause of the SBS40 mutation was previously unclear, our results suggest that this mutation is induced by γ-ray irradiation.

### 3.2 Radiation-associated increase in SV-independent SNVs

**Fig. 2:**
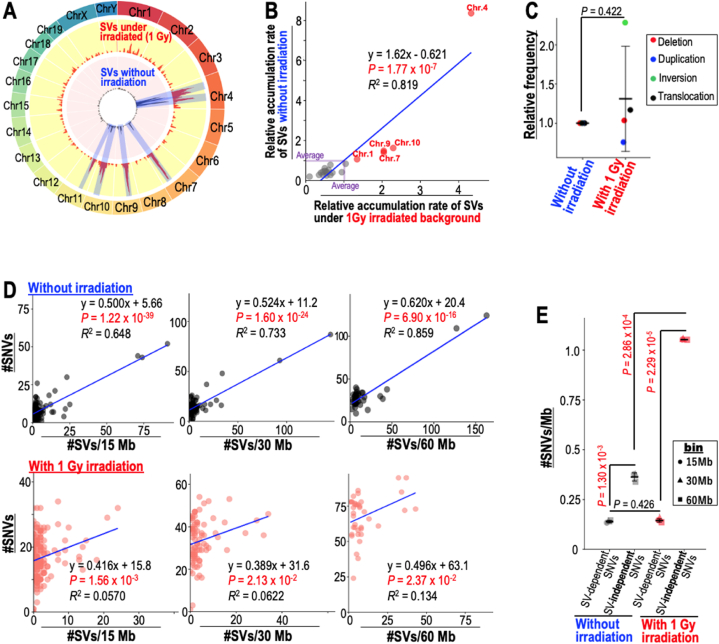
**SV-independent SNVs induced by radiation. (A)** SVs induced in unirradiated and irradiated immortalized MEFs were analyzed using circos plots and compared. Chromosome ideograms are shown around the outer ring. The two inner circular tracks show the numbers of SVs induced in unirradiated MEFs (black in pink background) and the number of SVs induced in irradiated MEFs (red in yellow background). Hotspots of SV-induction sites are marked with a blue shadow. **(B)** Correlations between SVs induced in unirradiated and those induced in irradiated MEFs were evaluated together with the accumulation rates of SVs in each chromosome. Student's *t*-test was used for statistical analysis. **(C)** Relative rates of each type of genomic alteration in unirradiated and irradiated MEFs were analyzed and compared after normalized with chromosomal numbers. Two-tailed Welch's *t*-test was used for statistical analysis. **(D,E)** To confirm the presence of SV-dependent and -independent SNVs, correlations between SVs and SNVs were evaluated after their numbers in every 15, 30, and 60 Mb window had been counted. **(D)** SV-dependent and -independent SNVs were compared in unirradiated and irradiated MEFs. Two-tailed Student's *t*-test and Welch's *t*-test were used for statistical analysis in **(D)** and **(E)**. (For interpretation of the references to colour in this figure legend, the reader is referred to the Web version of this article.)

A previous study revealed that genomic instability after γ-ray irradiation is due to replication stress-associated DSBs [7,8], which is also responsible for genomic instability even in the absence of irradiation [11,12]. In fact, we observed that SV hotspots were identical in unirradiated and irradiated immortalized MEFs (Fig. 2A; see hotspots marked with a blue shadow). We further compared SV induction in the irradiated and unirradiated backgrounds and observed that the rates of SV accumulation in each chromosome were tightly correlated between those two backgrounds (Fig. 2B). These results indicate that SV-induction sites are basically not affected by γ-ray irradiation. In addition, γ-ray irradiation did not affect the number of SVs when their number was normalized with chromosome numbers (Fig. 2C). Thus, although radiation exposure is associated with an increased risk of genomic instability because of the accumulation of replication stress-associated DSBs [8], the resulting SV-induction positions and numbers are not affected, implying that genomic regions subjected to replication stress are likely not to be affected by γ-ray irradiation.

To clarify the SV-dependent and -independent SNVs, we further analyzed the association between SVs and SNVs, in which the numbers of SV-dependent and -independent SNVs were estimated as described in Materials and Methods. Since our previous study revealed that an association between SVs and SNVs is observed when the analysis is done over wide genomic regions, i.e., 0.1–50 M base ranges [8], we examined whether this occurred in several genomic windows (i.e., 15, 30, 60 M bases), which yielded results showing that the association was independent of window size (Fig. 2D). Importantly, we observed that the number of SV-independent SNVs increased after irradiation. While the number of SV-dependent SNVs was not affected by γ-ray irradiation, SV-independent SNVs were increased 2.8-fold by irradiation (Fig. 2E). Together with the results shown in Fig. 1, we concluded that the risk of inducing SNVs by irradiation is different from the risk of inducing SVs by irradiation. These results imply that the SNVs induced by γ-ray irradiation are mainly induced during canonical replication, unlike SNVs associated with SVs.

### 3.3 Increased ROS after γ-ray irradiation

**Fig. 3:**
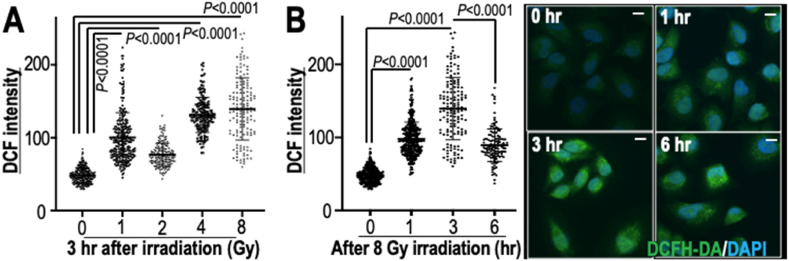
**Increase in ROS after irradiation. (A,B)** The ROS level was measured using the DCF assay at different γ-ray irradiation doses **(A)** and hours after 8 Gy irradiation **(B)**. Bars in the graph show means ± s.d. Statistical analyses were performed by two-tailed Welch's t-tests. Scale bars in images, 10 μm.

Since ROS are a possible cause of SBS17a and 17b signatures, we next tested ROS levels after radiation exposure using 2,7-Dichlorodihydrofluorescein diacetate (DCFHDA), which is immediately oxidized to 2,7-Dichlorofluorescein (DCF) after exposure to ROS. As expected, the ROS level detected by DCF increased after irradiation (Fig. 3A). In addition, ROS continued to increase for several hours after irradiation and then decreased (Fig. 3B). Although it is still unclear how ROS are induced by γ-ray irradiation, the decrease in ROS several hours after irradiation suggests the oxidation of bio-molecules by ROS, which could include nucleotide oxidation. In fact, multiple types of oxidized nucleotide adducts have been widely reported to be associated with high ROS levels, and these oxidized nucleotide adducts further increase the risk of SNV induction [22].

### 3.4 SBS17a and 17b signatures occur in radiation-associated human cancer

**Fig. 4:**
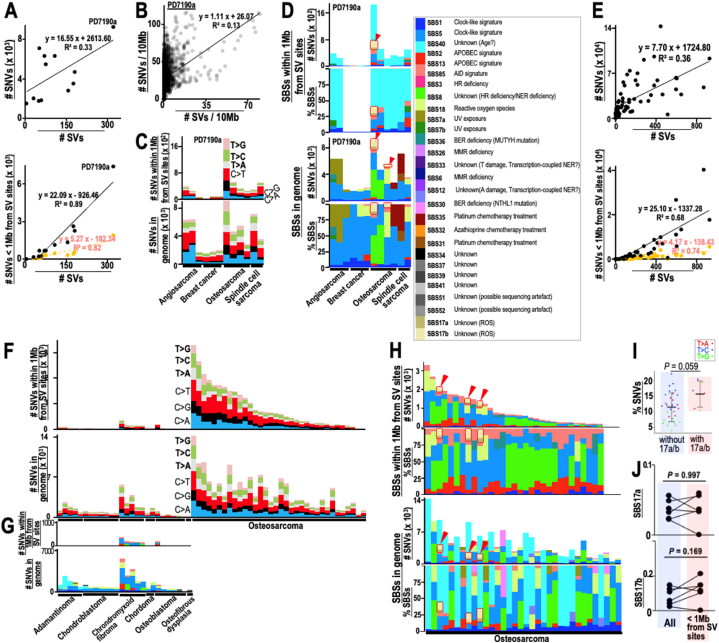
**SV-independent induction of SBS17a and 17b in the human cancer genome. (A**–**D)** SVs, SNVs, and SBS signatures in 12 radiation-associated secondary malignancies were analyzed. Associations between SVs and SNVs was analyzed in 12 radiation-associated secondary malignancies **(A,** top panel**)**. SNV numbers within 1 Mb of SV sites were analyzed in each case and compared with those expected on a random basis **(A,** bottom panel**)**. Correlations between SV and SNV numbers in each tumor were plotted in 10 Mb windows **(B)**. SNV types induced in each case were analyzed within 1 Mb of SV sites and in the whole genomes **(C)**. SBS signatures induced in 12 radiation-associated secondary malignancies were analyzed within 1 Mb of SV sites and in whole genomes **(D)**. The fraction of SBS17a and 17b signatures is shown with red arrowheads. **(E**–**H)** SVs, SNVs, and SBS signatures in bone tumors were analyzed. Association between the generation of SVs and SNVs was analyzed in bone tumors **(E,** top panel**)**. SNV numbers within 1 Mb of SV sites were analyzed in each case and compared with those expected on a random basis **(E,** bottom panel**)**. SNV types induced in each case were analyzed within 1 Mb of SV sites and in whole genomes **(F)**. SBS signatures induced in bone tumors were analyzed within 1 Mb of SV sites and in whole genomes **(G)**. The fraction of SBS17a and 17b signatures is shown with red arrowheads **(H)**. **(I)** The fractions of each SNV type in A-T bps were compared between tumor cases with and without SBS17a and 17b signatures. Statistical analyses were performed by two-tailed Welch's t-tests. **(J)** Levels of SBS17a and 17b signatures within 1 Mb of SV sites were compared with those in the whole genomes. Statistical analyses were performed by two-tailed Welch's t-tests. (For interpretation of the references to colour in this figure legend, the reader is referred to the Web version of this article.)

Our results revealed that SV-independent induction of SBS17a, 17b, and 40 after γ-ray irradiation was associated with increases in ROS levels. To study the mutational SBS signatures of human cancers that occur in association with ionizing radiation, we next focused on 12 previously reported radiation-associated secondary malignancies [19]. In the 12 cases, SNV levels were tightly correlated with SV levels (Fig. 4A top panel), and SNV numbers around SV sites (<1 Mb) were higher than expected on a random basis (Fig. 4A bottom panel). The numbers of these SNVs were high in regions with high numbers of SVs (Fig. 4B; see PD7190a showing the highest SNV numbers). Since SNVs can be induced in different therapeutic backgrounds, the resulting SNV levels varied (Fig. 4C) and hence the signatures were complex (Fig. 4D). In fact, while the clock-like signatures SBS1 and 5 were present as major signatures in the genome, the other signature types varied, including chemotherapy-associated signatures SBS31 and 35, HR deficiency-associated signature SBS3, and the APOBEC signature SBS13 (Fig. 4D). Importantly, despite these complications in the resulting signatures, SBS17a and 17b were still observed in PD7530a and PD7189a, both of which were osteosarcoma cases. These signatures did not accumulate around SV sites, supporting SV-independent induction of these signatures. By contrast, SBS40 was only observed around SV sites, implying that SBS40 in those cancer genomes is different from that in cells irradiated *in vitro*. In fact, SBS40 was also observed around SV sites even *in vitro* without irradiation (Fig. 1D).

We further analyzed the statuses of SBS signatures and their dependence on SVs in another bone tumor cohort (Fig. 4E–H), because SBS17a and 17b were observed in bone tumor osteosarcoma (Fig. 4D). These SNVs were again tightly associated with SVs and accumulated strongly around SV sites (Fig. 4E). SNV levels and the signatures observed in these tumors varied (Fig. 4F–H); SBS17a and 17b signatures were observed in three out of 34 osteosarcoma cases (Fig. 4G and H: see signals indicated by red arrow heads). This indicates that γ-ray irradiation is not the only cause of the SBS17a and 17b signatures.

As SNVs in irradiated cells were enriched in A-T bps in association with SBS17a and 17b signatures (Fig, 1B), SNV levels in A-T bps were analyzed and compared between those with and without SBS17a/17b. Indeed, similar to results *in vitro*, SNVs induced in A-T bps in SBS17a/17b-positive cases were present in higher numbers than those in SBS17a/17b-negative cases, even in those human patient cohorts, although the difference was not statistically significant (Fig. 4I). In addition, we also compared the levels of SBS17a and 17b signatures around SV sites with those in the whole genome, and observed identical levels of these signatures (Fig. 4J). This indicates that the risk of inducing SBS17a and 17b signatures is separate from that of inducing SVs even in human cancer cells.

## 4 Discussion

Our results revealed that radiation exposure is associated with an increased risk of SNV induction, which was especially associated with the mutational signatures SBS17a, 17b, and 40. Such SNV induction is associated with oxidation, which is supported by the following findings: (1) SBS17a and 17b signatures were associated with the ROS that accumulated in the irradiated cells; (2) the ROS level was elevated after irradiation; (3) the ROS level subsequently decreased, which is an expected consequence of bio-molecule oxidation. Unlike many other SNVs, SBS17a/17b/40-associated SNVs are separately induced from SVs, although minor SV-associated SNV induction is still possible. These results suggest that these SNVs are mainly induced during canonical replication, unlike the SNVs associated with SVs.

Although γ-ray irradiation is associated with a particular type of SNV induction, which especially targets the middle T in the XTT sequence context, it is still unclear what causes SNV levels to increase. Our results suggest this is due to increased ROS levels following γ-ray irradiation. However, the major SNVs induced by γ-ray irradiation are not thought to be caused by 8-oxo-guanine, i.e., a major oxidized nucleotide that leads to SNVs, because the SNVs caused by 8-oxo-guanine are mainly C > A transversions [23], while the SNVs induced by irradiation are mainly T > G, T > C, and T > A transversions (Fig. 1A). One possible explanation is that other nucleotide adducts are involved. In fact, many different types of nucleotide adducts are induced by oxidation [24], among which 8-oxo-guanine is repairable by BER [25]. Another possibility is the involvement of species other than ROS such as reactive nitrogen species (RNS). In that event, then ROS elevation might be a secondary effect of γ-ray irradiation. These possibilities could be addressed by performing experiments with antioxidants.

Hypermutation is associated with a higher risk of cancer, as observed for mismatch repair deficient cells [12,26,27] and pol ε [28,29]. Therefore, SNVs caused by γ-ray irradiation could increase cancer risk together with SV-associated chromosomal alterations. An important question is what primarily increases the risk of carcinogenesis, ROS-associated SNVs or SV-associated chromosomal alterations. SV-associated chromosomal alteration is a strong possibility, because cancer is induced by loss of function of cancer-suppressor genes, which is usually induced by mutations in both alleles of these genes. Although mutations in both alleles of cancer-suppressor genes occur rarely as a result of simple replication errors, they can occur through loss of heterozygosity (LOH), i.e., caused by erroneous HR between allelic chromosomes. Supporting this conjecture, clonal evolution of MEFs mutated in the ARF/p53 pathway occurs together with massive SV induction and the resulting mutations are found in both alleles, but they are effectively suppressed as long as genome stability is maintained [12].

Unlike SBS signatures in the MEF model, human cancer signatures are complex, probably because each patient has their own particular lifestyle and medical and therapeutic histories differ between patients. In fact, complex signatures include chemotherapy-associated signatures and APOBEC signatures that arise in a background of increased inflammation. These results suggest that, in human, SNVs of multiple etiologies are associated with a higher risk of radiation-associated secondary malignancies.

## Data availability

All data generated or analyzed during this study are included in this published article and its supplementary information files or referenced in this article.

## Additional information

No additional information is available for this paper.

## CRediT authorship contribution statement

**Yuya Manaka:** Writing – review & editing, Visualization, Validation, Methodology, Investigation, Formal analysis, Conceptualization. **Rika Kusumoto-Matsuo:** Writing – review & editing, Visualization, Validation, Methodology, Investigation, Formal analysis. **Yusuke Matsuno:** Writing – review & editing, Visualization, Validation, Methodology, Investigation. **Haruka Asai:** Writing – review & editing, Visualization, Validation, Investigation, Formal analysis. **Ken-ichi Yoshioka:** Writing – review & editing, Writing – original draft, Supervision, Resources, Project administration, Funding acquisition, Conceptualization.

## Declaration of competing interest

The authors declare that they have no known competing financial interests or personal relationships that could have appeared to influence the work reported in this paper.
